# Polyamine flux suppresses histone lysine demethylases and enhances *ID1* expression in cancer stem cells

**DOI:** 10.1038/s41420-018-0117-7

**Published:** 2018-11-13

**Authors:** Keisuke Tamari, Masamitsu Konno, Ayumu Asai, Jun Koseki, Kazuhiko Hayashi, Koichi Kawamoto, Noriyuki Murai, Senya Matsufuji, Fumiaki Isohashi, Taroh Satoh, Noriko Goto, Shinji Tanaka, Yuichiro Doki, Masaki Mori, Kazuhiko Ogawa, Hideshi Ishii

**Affiliations:** 1 Department of Radiation Oncology, Osaka University Graduate School of Medicine, Osaka, 565-0871 Japan; 2 Department of Frontier Science for Cancer and Chemotherapy, Osaka University Graduate School of Medicine, Osaka, 565-0871 Japan; 3 Department of Medical Data Science, Osaka University Graduate School of Medicine, Osaka, 565-0871 Japan; 4 Division of Hospital, National Institute of Radiological Sciences, Chiba, 263-8555 Japan; 5 Department of Gastroenterological Surgery, Osaka University Graduate School of Medicine, Osaka, 565-0871 Japan; 6 Department of Molecular Biology, Jikei University School of Medicine, Tokyo, 105-8461 Japan; 7 Cancer Research Institute, Kanazawa University, Kanazawa, 920-1192 Japan; 8 Department of Molecular Oncology, Tokyo Medical and Dental University, Tokyo, 113-8510 Japan

## Abstract

Cancer stem cells (CSCs) exhibit tumorigenic potential and can generate resistance to chemotherapy and radiotherapy. A labeled ornithine decarboxylase (ODC, a rate-limiting enzyme involved in polyamine [PA] biosynthesis) degradation motif (degron) system allows visualization of a fraction of CSC-like cells in heterogeneous tumor populations. A labeled ODC degradation motif system allowed visualization of a fraction of CSC-like cells in heterogeneous tumor populations. Using this system, analysis of polyamine flux indicated that polyamine metabolism is active in CSCs. The results showed that intracellular polyamines inhibited the activity of histone lysine 4 demethylase enzymes, including lysine-specific demethylase-1 (LSD1). Chromatin immunoprecipitation with Pol II antibody followed by massively parallel DNA sequencing, revealed the global enrichment of Pol II in transcription start sites in CSCs. Increase of polyamines within cells resulted in an enhancement of ID1 gene expression. The results of this study reveal details of metabolic pathways that drive epigenetic control of cancer cell stemness and determine effective therapeutic targets in CSCs.

## Introduction

Recent advances in understanding tumor heterogeneity have revealed the presence of subpopulations of highly tumorigenic cancer stem cells (CSCs) and weakly tumorigenic non-CSCs^1^. Compared with non-CSCs, CSCs possess tumorigenic, self-renewal, and multilineage differentiation potential and are resistant to chemotherapeutic agents and radiotherapy, so they cause treatment failure due to tumor recurrence and metastases^1^.

Researchers have studied differences between metabolic activities in CSCs and non-CSCs mainly through glucose metabolism^2^. Non-CSCs depend on glycolysis for survival and growth, whereas CSCs rely heavily on both glycolysis and oxidative phosphorylation (OXPHOS)^3,4^. In addition, the biological behavior of cancer cells involves methylation of histones, RNA, and DNA, all of which are modulated epigenetically by S-adenosylmethionine (SAM), a methyl-donating compound^5^. Locasale found that one-carbon metabolism, comprising three reactions (folate cycle, methionine cycle, and transsulfuration pathway), couples with SAM generation and fuels polyamine (PA) metabolism^6^. However, despite these and other studies, the differences between PA metabolism in CSCs and non-CSCs are not entirely understood. PAs include putrescine, spermidine, and spermine and play an essential role in cell proliferation, cell survival, and cancer progression^7^.

Ornithine decarboxylase (ODC), as a rate-limiting enzyme, converts ornithine to putrescine as the first step in intracellular PA biosynthesis^7^. ODC is degraded by proteasomes, depending on its unique degradation motif (degron) but independent of ubiquitylation^8^. A study of the *Zoanthus* sp. green fluorescent protein (ZsGreen)–degron^ODC^ fusion system enables visualization of a small population of tumorigenic CSCs^9^.

The maintenance of CSC “stemness” and differentiation to non-CSCs is controlled by epigenetic mechanisms^10–12^. PAs work potentially as epigenetic regulators, and previous studies on PAs and histone acetylation have shown their involvement in transcription and gene expression control^13^. PAs are positively charged molecules and thus can interact with negatively charged DNA and RNA^14^. Subsequent changes in the chromatin structure can affect gene transcription, cell proliferation, and cell differentiation, suggesting that PA flux plays a role in chromatin remodeling and cell proliferation.

Lysine-specific demethylase-1 (LSD1), a nuclear homolog of amine oxidases, demethylates histone H3 lysine 4 (H3K4) to close chromatin for transcriptional silencing^15^. LSD1 is overexpressed in several cancers, such as bladder, lung, pancreatic, and cervical cancers and neuroblastoma^16–20^. Polyamine oxidase (PAOX) converts spermine to spermidine and spermidine to putrescine^7^. The structure of PAOX’s catalytic pocket resembles LSD1’s enzyme pocket^21,22^. Many studies have been conducted to discover drugs targeting LSD1 using PA analogs^23,24^. However, it is still unclear how natural PAs inhibit LSD1, how they control epigenetics in CSCs, and what results from chromatin modification. We studied the effect of PA flux using the ZsGreen–degron^ODC^ fusion system and demonstrated that PA flux increase in CSCs modulates LSD1 function and remodels the expression of stemness genes, such as *ID1*, which further augment the tumorigenic nature of CSCs.

## Results

### The critical role of the ODC protein and polyamine flow in CSCs

**Fig. 1:**
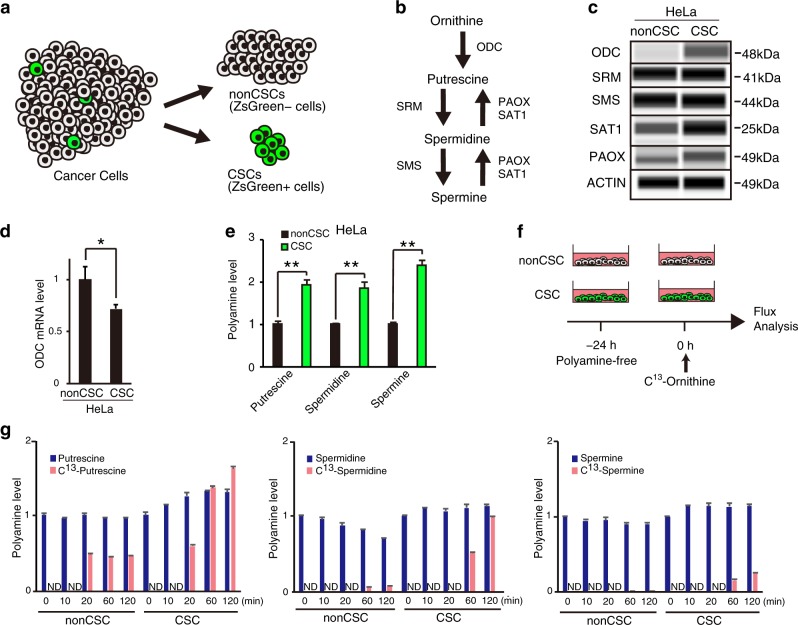
CSCs have high ODC and PA levels and rapidly convert them from ornithine to PAs. **a** A method of identifying CSCs and non-CSCs using the ZsGreen–degron^ODC^ fusion system. CSCs are identified as ZsGreen-positive cells, whereas non-CSCs are identified as ZsGreen-negative cells. **b** Schema of PA metabolism. **c** Immunoblot for ODC, SRM, SMS, SAT1, PAOX, and actin using HeLa CSCs and non-CSCs. See also Figure S1 for immunoblot for other cell lines. **d** Expression of the *ODC* gene was examined (*n* = 3) by qPCR. Data are represented as mean ± SD. *p < 0.05. **e** Intracellular putrescine, spermidine, and spermine levels of HeLa CSCs and non-CSCs examined by GC-MS analysis (*n* = 3). Data are represented as mean ± SD. **p < 0.01. **f** Scheme of experimental design for C^13^-ornithine flux analysis. Before adding C^13^-ornithine, 24 h incubation with PA-free medium was performed for PA depletion. **g** C^13^-ornithine flux analysis by GC-MS with HeLa cells collected at 0, 10, 20, 60, and 120 min. Data are normalized to concentrations of normal PAs at 0 min and represented as mean ± SD (*n* = 2). ND, not detected

To construct the ZsGreen–degron^ODC^ fusion system, we used retroviral-mediated gene transfer of the green fluorescent protein–fused ODC degron in cancer cells^9,25–27^, enabling visualization of a CSC population as ZsGreen-positive cells (Fig. 1a). Immunoblots using an anti-ODC antibody showed that protein levels of ODC, spermidine/spermine *N*1-acetyltransferase (SAT1), and PAOX were high in CSCs in cervical cancer and osteosarcoma cell lines (see chemistry in Fig. 1b and results in Fig. 1c, S1a). The expression of other PA-metabolizing enzymes, such as spermidine synthase (SRM) and spermine synthase (SMS), showed little difference between CSCs and non-CSCs, as determined using specific antibodies (Fig. 1c, S1a). ODC messenger RNA (mRNA) expression levels determined by real-time quantitative polymerase chain reaction (Fig. 1d) were not high. This result is supported by previous studies indicating that ODC accumulates by suppression of ubiquitin-independent degradation in proteasomes^9,25–27^. The data suggest that CSCs express a high level of ODC protein because of slow degradation and not because of ODC production increase.

Out findings prompted us to investigate PA metabolism in a ZsGreen-positive CSC population of HeLa cells. We measured the concentration of cellular PAs by gas chromatography–mass spectrometry (GC-MS). The results showed a 1.9-fold increase in PA concentration ratios of CSCs/non-CSCs in putrescine (*p* < 0.01), a 1.9-fold increase in spermidine (*p* < 0.01), and a 2.4-fold increase in spermine (*p* < 0.01), suggesting that PA levels increased in CSCs compared with non-CSCs (Fig. 1e).

Next, we studied PA flux. CSCs and non-CSCs were starved extracellular PA in a medium supplemented with dialyzed serum, exposed to ^13^C-ornithine, and performed GC-MS. The results showed that ornithine-to-PA conversion within 120 min was faster in CSCs than in non-CSCs (Fig. 1f, g), whereas the concentration of spermine was higher in non-CSCs over 12 h after addition of ^13^C-ornithine to the culture (Fig. S1b). These results suggested that CSCs possess higher levels of cellular PAs, which are maintained by rapid conversion of ornithine to putrescine, spermidine, and spermine, as noted by early time stimulation after starvation; the results were consistent with a mathematical study of PA flow in CSCs^28^.

### Inhibition of LSD1 activity by polyamines

**Fig. 2:**
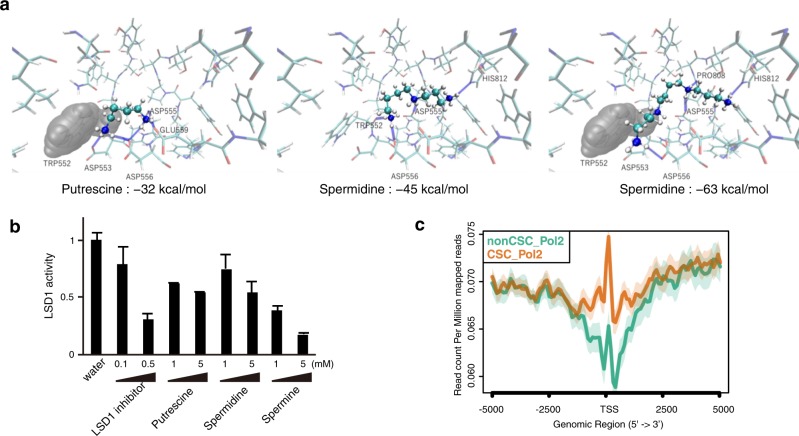
PAs can inhibit H3K4 demethylases. **a** Molecular docking simulation. Putrescine, spermidine, and spermine can inhibit the H3K4-demethylase site of LSD1. The binding free energies (Δ*G*) between PAs and LSD1 are described. Hydrogen bonds were shown as blue dotted lines. van der Waals forces were also shown between PAs and LSD1. **b** Enzymatic activity assay of LSD1. Demethylase activity was normalized by water (control). Data are represented as mean ± SD (*n* = 3). **c** Pol II peaks shown as a TSS plot produced by ChIP-Seq of HeLa CSCs and non-CSCs. ChIP-seq data are deposited at Gene Expression Omnibus (GEO), accession number GSE103187

We suspected that cellular PA increase induces a CSC-like phenotype. Given that LSD1 plays an important role in stemness or pluripotency maintenance in human embryonic stem cells^29^, we assumed that PAs may inhibit LSD1 demethylase sites and modulate epigenetics, resulting in stem phenotype induction in cancer cells. Computational structure analysis performed to this end showed that PAs bind to the LSD1 demethylase site. Binding energies between the LSD1 demethylase site and PAs were −32 kcal/mol for putrescine, −45 kcal/mol for spermidine, and −63 kcal/mol for spermine, suggesting that among all PAs, spermine has the strongest binding (Fig. 2a). The enzyme assay also showed that putrescine, spermidine, and spermine inhibit LSD1 activity at 1 and 5 mM in a dose-dependent manner and that spermine is the strongest inhibitor (Fig. 2b). Examination of the cytotoxic effects of PAs showed that spermine is the most cytotoxic (Fig. S2a). These results suggested that PAs can inhibit LSD1 activity. In addition, further examination indicated that the demethylase activity of other histone demethylases, such as JMJD2A and KDM5B, is inhibited by PAs (Fig. S2b, c).

Next, we performed ChIP-Seq analysis of CSCs and non-CSCs in order to study polymerase II antibodies (Pol II) interactions across the entire genome. The results showed that Pol II is enriched in transcription start site (TSS) in CSCs compared with non-CSCs (Fig. 2c).

We concluded that PAs inhibit LSD1 activity and induce transcriptional modifications via epigenetic H3K4 demethylation.

### *ID* gene expression induction by polyamines

**Fig. 3:**
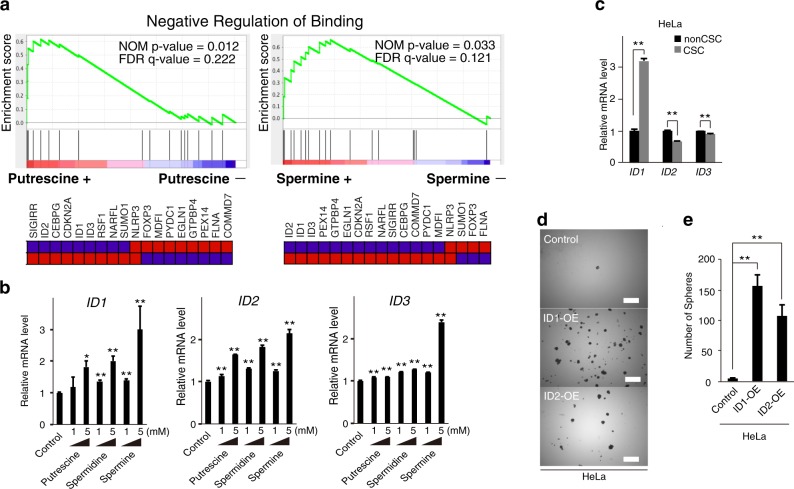
Gene expression profiles in HeLa cells exposed to PAs and overexpression of *ID* genes induces cancer cell stemness. **a** Gene set enrichment analysis by 5 mM putrescine and 5 mM spermine exposure. Microarray data are deposited at GEO, accession number GSE102052 and GSE102053. **b** Expression of *ID1*, *ID2*, and *ID3* genes (*n* = 3) examined by qPCR. *p < 0.05; **p < 0.01. **c** Expression of *ID1*, *ID2*, and *ID3* genes (*n* = 3) in HeLa CSCs and non-CSCs examined by qPCR. Data are represented as mean ± SD. *p < 0.05. **d** Representative images of the sphere formation assay using HeLa control, ID1-OE, and ID2-OE cells. **e** Sphere formation assay using HeLa control, ID1-OE, and ID2-OE cells. Data are represented as mean ± SD. **p < 0.01

To study the gene expression profile induced by cellular PA increase, we performed microarray analysis of HeLa cells exposed to putrescine and spermine. We used gene set enrichment analysis to interpret gene expression data and found that the gene set *negative regulation of binding* significantly changed in cells exposed to putrescine and spermine, including expression of *inhibitors of differentiation*, *ID1*, *ID2*, and *ID3* (Fig. 3a, b). Previous reports indicate that ID family members play a role in promoting malignant biological phenotypes in cancer^30^. Consistent with earlier studies, this study indicated that PA increase is associated with up-regulation of *ID1*, *ID2*, and *ID3* mRNA expression and therefore inhibition of cancer cell differentiation.

### Overexpression and self-renewal induction of *ID1* in CSCs

Next, we examined *ID* gene expression in the ZsGreen–degron^ODC^ fusion system. The data indicated that *ID1* is overexpressed in CSCs (Fig. 3c). To investigate the role of *ID1* in CSC-like phenotypes, we studied overexpression of *ID1* in HeLa (HeLa-ID1-OE) cells. Both cell types showed higher sphere-forming capacity compared with control cells. However, studies of *ID2* overexpression in HeLa cells showed higher sphere-forming capacity in ID2-OE cells than in control cells (Fig. 3d, e). These results suggested that the ID family has the potential to enhance cancer cell stemness.

**Fig. 4:**
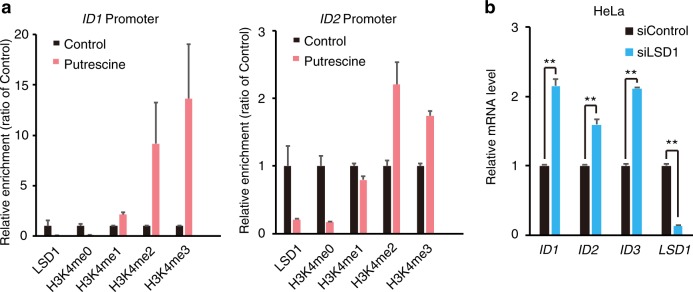
PA inhibits LSD1 and results in H3K4-hypermethylation on *ID1* and *ID2* promoters. **a** PCR analysis of *ID1* and *ID2* promoters after ChIP experiments with anti-LSD1 and H3K4 me0, H3K4 me1, H3K4 me2, and H3K4 me3 antibodies in HeLa cells exposed to 5 mM putrescine for 24 h. Data were normalized to be a ratio of the control value from each experiment and are represented as mean ± SD. **b** Expression of *ID1*, *ID2*, *ID3*, and *LSD1* genes in the siControl and siLSD1 in HeLa cells (*n* = 3) examined by qPCR. Data are represented as mean ± SD. **p < 0.01

### Epigenetic upregulation of *ID* genes by polyamines

**Fig. 5:**
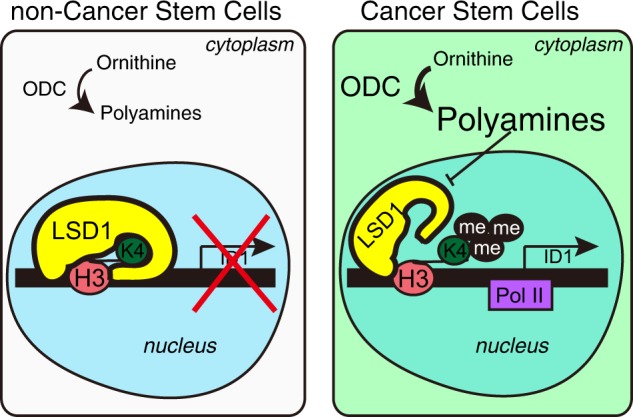
Schema of epigenetic control of ID1 by PAs in CSCs. Comparing with non-CSCs, CSCs have considerable ODC protein which activates PA metabolism. Elevated PAs inhibit histone H3K4 demetylase LSD1 and epigenetically induce ID1 expression. ID1 is essential gene for cancer cell stemness

To confirm *ID* gene epigenetic regulation, we carried out ChIP-PCR of the *ID* gene promoter region by H3K4 methylation antibodies and LSD1 antibody. The results showed that an increase in PAs, especially putrescine, induces H3K4 methylation and inhibition of LSD1 binding of promoter regions (Fig. 4a). In addition, to examine whether LSD1 function decrease causes an increase in *ID* gene expression, we performed LSD1 knockdown in HeLa cells by small interfering RNA (siRNA), inducing *ID* gene expression (Fig. 5b). These results suggested that PAs, mainly putrescine, inhibit LSD1 function and stimulate high levels of methylated H3K4 in regulatory regions of *ID* gene expression.

## Discussion

In this study, we showed that CSCs have a higher level of PAs compared with non-CSCs. The reason is presumably ODC function, as demonstrated by the pulse-chase experiment, in which CSCs showed higher PA metabolism compared with non-CSCs, using the ZsGreen–degron^ODC^ fusion system. Our results suggested that ODC protein levels are upregulated via a post-transcriptional mechanism. ODC is regulated via many processes, for example, ubiquitin-independent degradation by the 26S proteasome and degradation by antizyme through frame-shift-dependent translational regulation^31^. Our hypothesis was that in CSCs, the PA production mechanism might be established in the ODC pathway, which was relevant to a CSC-like nature, as shown in our ZsGreen–degron^ODC^ fusion system. To the best of our knowledge, ours is the first report on CSCs expressing substantial ODC levels and PAs. Intracellular PAs and ODC play a role in cancer progression^7^. For example, in hedgehog-dependent medulloblastoma, ODC and PA levels are elevated and pharmacological inhibition of PA axis efficiently blocks medulloblastoma cell proliferation^32^. In some cancers with poor prognosis, ODC is overexpressed^33,34^.

We showed that in the ZsGreen–degron^ODC^ fusion system for CSC identification, increased PAs in CSCs inhibit H3K4-specific demethylases, such as LSD1 and JARID1B. It has been proposed that increased PAs act as antagonists to H3K4 enzymatic demethylation, which was more apparent in CSCs than in non-CSCs. As H3K4 methylation enhances transcriptional activation^35^, PA-flux-induced LSD1 inhibition may contribute to induction of CSC-like phenotypes.

ID proteins are transcriptional regulators that control differentiation in stem and progenitor cells^30^. In cancer, ID proteins are induced by oncoproteins (e.g., MYC, RAS, SRC, Notch, EWS-FLI1, and receptor tyrosine kinases) and growth factor-directed signals (e.g., epidermal growth factor [EGF], basic fibroblast growth factor [bFGF], transforming growth factor-β, and bone morphogenetic proteins)^30^. However, little is known about the epigenetic control of *ID* genes with regard to histone modification. This study demonstrated that increased PA levels induce H3K4 methylation and trigger *ID1* transcription in CSCs. Considering that high levels of ID protein expression in cancer cells are important, potential prognostic and diagnostic markers in several tumors, including breast, colorectal, liver, and prostate cancers^36–39^, the treatment strategy of targeting ID1 in CSCs is a promising way to cure cancer.

## Materials and methods

### Cell culture

Human cervical cancer cell lines (HeLa, CaSki, and ME180) and osteosarcoma cell lines (MG63 and U2OS) were purchased from the American Type Culture Collection (VA, USA) and were cultured in Dulbecco’s modified Eagle’s medium (DMEM) supplemented with 10% fetal bovine serum (FBS), 100 U/mL penicillin, and 100 µg/mL streptomycin.

### Stable cell lines

The retroviral expression vector pQCXIN-ZsGreen-cODC, in which the C-terminal region (37 amino acids) of murine ornithine decarboxylase (termed “cODC” or “degron”) was fused to ZsGreen, was used to visualize the CSC population as ZsGreen-positive cells. The proteasome sensor vector ZsProSensor-1 (632425, Clontech, CA, USA) encodes the gene for ZsGreen-cODC, and ZsGreen-cODC was digested with BglII and NotI from ZsProSensor-1 and cloned into pQCXIN (631514, Clontech).

To construct ID1 and ID2 overexpression vectors, ID1 and ID2 ORF sequences were amplified by PCR with the primers described in Table S1. Then, these PCR products were digested with NotI and XhoI and then cloned into pMXs-IRES-Neo.

To generate a retrovirus, each retroviral vector was transfected into platinum A (Plat-A, RV-102, Cell Biolabs) retroviral packaging cells using FuGENE 6 (E2691, Promega, WI, USA). After 24 h incubation, the virus collected from the Plat-A supernatant was used to infect osteosarcoma and cervical cancer cells. Stable transfectants were selected with 200 μg/mL of Geneticin (10131027, Thermo Fisher Scientific, MA, USA) in a culture medium for 2 weeks.

### Polyamine deprivation and exposure

For PA deprivation, we used DMEM supplemented with 10% dialyzed FBS (26400044, Thermo Fisher Scientific), 100 U/mL penicillin, and 100 µg/mL streptomycin. For PA exposure, 3 and 5 mM putrescine (P5780, Sigma-Aldrich, MO, USA), spermidine (S0266, Sigma-Aldrich), and spermine (S4264, Sigma-Aldrich) were added directly to DMEM supplemented with 10% dialyzed FBS, 100 U/mL penicillin, 100 µg/mL streptomycin, and 1 mM aminoguanidine hydrochloride (396494, Sigma-Aldrich). Aminoguanidine was routinely added to inhibit serum amino-oxidases and prevent extracellular PAs toxicity due to reactive oxygen species (ROS) generation^40^

### Sphere formation assay

Cells were plated separately, with 3000 cells on ultralow-attachment six-well plates (3471, Corning, NY, USA), and incubated in serum-free DMEM/F12 medium (11330032, Thermo Fisher Scientific) supplemented with 20 ng/mL bFGF (F0291, Sigma-Aldrich), 20 ng/mL EGF (E9644, Sigma-Aldrich), and N-2 MAX media supplement (AR009, R&D Systems, MN, USA). After 14 days, spheres with diameters >100μm were counted.

### Quantification of intracellular polyamines

We sorted one million non-CSCs and CSCs by fluorescence-activated cell sorting (FACS) and centrifuged at 1000 × *g* for 10 min at 4 °C. Cell pellets were washed twice with phosphate-buffered saline (PBS). The washed pellets were homogenized on ice with 10% NaCl solution at pH 1 with HCl and then centrifuged at 12,000 × *g* for 15 min at 4 °C. The supernatant was extracted with 3 mL of diethyl ether by vortexing for 10 min and separated by centrifugation at 12,000 × *g* for 20 min, and the aqueous phases were collected. For N-ethoxycarbonylation of the amines, 1 mL of diethyl ether containing 50 μL of ethyl chloroformate was added to the sample solution. The reaction mixture was shaken at 23 °C for 30 min and centrifuged at 1200 × *g* for 5 min. The ether layer containing PA N-ethoxylcarbonyl (N-EOC) derivatives was transferred to a separate glass vial. This derivatization reaction was repeated by re-extracting the aqueous phase. The ether layers from the two extractions were combined and evaporated to complete dryness under a dry nitrogen stream. The dried PA N-EOC derivatives were put in 100 μL of ethyl acetate, to which 200 μL of trifluoroacetic anhydride was added. The sealed vials containing the mixture were placed on a 75 °C heating block for 1 h to complete the trifluoroacetylation. The mixture was then evaporated to complete dryness under a dry nitrogen stream. The derivatives were reconstituted in 200 μL of ethyl acetate, and 2 μL of aliquots were injected for GC-MS analysis in triplicate.

### Molecular docking simulation

To predict the PA docking poses, we performed docking simulations with Glide^41^ in Schrodinger Suite 2009 (Schrödinger, LLC, NY, USA). The binding poses for ornithine, putrescine, spermidine, and spermine were generated in Glide SP (standard precision) mode. The binding free energy (Δ*G*) for each pose was estimated with the molecular mechanics energies combined with the generalized Born and surface area continuum solvation (MM/GBSA) method using Prime^42^ in Schrodinger Suite 2009 (Schrödinger, LLC). For each ligand, we selected binding poses with the lowest MM/GBSA score. In the docking simulations, we took the LSD1 protein coordinate (PDB ID: 2H94) from the Protein Data Bank (PDB).

### Histone demethylase inhibition assays

For LSD1 and JMJD2A, we performed assays with 96-well plates. We added 120 μL of Enzyme Assay Buffer, 20 μL of LSD1 (or JMJD2A), 20 μL of horseradish peroxidase (HRP), 10 μL of fluorometric substrate, and 10 μL of solvent for 100% initial activity wells (positive control); 140 μL of Assay Buffer, 20 μL of LSD1 (or JMJD2A), 20 μL of HRP, 10 μL of fluorometric substrate, and 10 μL of solvent for background wells (negative control); and 120 μL of Assay Buffer, 20 μL of LSD1 (or JMJD2A), 20 μL of HRP, 10 μL of fluorometric substrate, and 10 μL of LSD1 inhibitor and PA solution for inhibitor wells. Then, we added 20 μL of peptide to all the wells, except background wells, incubated them for 30 min at 37 °C, and read the plates using an excitation wavelength of 530–540 nm and an emission wavelength of 585–595 nm.

For KDM5B, we performed the assay with 384-well plates. We added 2.5 μL of 4× HDM Assay Buffer, 3 μL of KDM5B, 1 μL of biotinylated substrate, 0.5 μL of water, and 3 μL of inhibitor buffer for 100% initial activity wells (positive control); 2.5 μL of 4× HDM Incomplete Assay Buffer, 3 μL of KDM5B, 1 μL of biotinylated substrate, 0.5 μL of water, and 3 μL of inhibitor buffer for background wells (negative control); and 2.5 μL of 4× HDM Assay Buffer, 3 μL of KDM5B, 1 μL of biotinylated substrate, 0.5 μL of water, 3 μL of inhibitor buffer, and 3 μL of PA solution for inhibitor wells. Then, we added 5 μL of Eu-labeled K4 antibody to all the wells and incubated them for 30 min at 23 °C, added 5 μL of dye-labeled acceptor to all the wells, and incubated them again for 1 h at 23 °C with shaking. Finally, we measured the fluorescent intensity at wavelengths of 620 and 665 nm in a microtiter-plate reader.

### Gene set enrichment analysis

We used gene set enrichment analysis to interpret gene expression data, as previously described^43^.

### Quantitative RT-PCR

Total RNA samples were extracted using ISOGEN reagent (311–02501, NIPPON GENE, Japan). To synthesize complementary DNA (cDNA), we used ReverTra Ace real-time quantitative polymerase chain reaction (RT-qPCR) Master Mix with genomic DNA remover (FSQ-301, Toyobo, Japan). We incubated 0.5 µg of total RNA for 5 min at 65 °C, mixed it with 4 × DN Master Mix, and incubated it for 5 min at 37 °C. Finally, we mixed 5 × RT Master Mix II and then incubated the mixture for 15 min at 37 °C, 5 min at 50 °C, and 5 min at 98 °C to obtain cDNA.

We performed RT-qPCR using LightCycler FastStart DNA Master SYBR Green I (12239264001, Roche Diagnostics, Mannheim, Germany) on a LightCycler 2.0 (Roche Diagnostics) according to the manufacturer’s instructions. We gently mixed 9.4 µL of water, 1.6 μL of LightCycler FastStart DNA Master SYBR Green I, 1 μL of forward primer (10 µM), and 1 μL of reverse primer (10 µM) by pipetting and then added 2 μL of cDNA. The mixture was transferred to a LightCycler capillary. PCR was performed by initial denaturation for 10 min at 95 °C, 45-cycle quantification (denaturation for 10 s at 95 °C, annealing for 10 s at 55–60 °C, and extension for 10 s at 72 °C), and melting curve (denaturation for 0 s at 95 °C, annealing for 15 s at 65 °C, and extension for 0 s at 72 °C). Primer sequences are listed in Table S1.

### siRNA

HeLa cells were seeded in 6-well plates (4.0 × 10^5^ cells/well) and transfected with 10 nM small interfering RNAs (siRNAs) in the presence of Lipofectamine RNAiMAX (13778150, Thermo Fisher Scientific). The following siRNAs were used: siLSD1 (SASI_Hs01_00213078, Sigma-Aldrich), sixCT#1 (SASI_Hs02_00345461, Sigma-Aldrich), sixCT#2 (SASI_Hs01_00158008, Sigma-Aldrich), and a negative control (SIC-001–5, Sigma-Aldrich).

### Immunoblotting

Sorted CSCs and non-CSCs were centrifuged, the supernatant discarded, and the cells washed with PBS twice. Radioimmunoprecipitation assay lysis buffer (89900, Thermo Fisher Scientific; 25 mM Tris•HCl pH 7.6, 150 mM NaCl, 1% NP-40, 1% sodium deoxycholate, 0.1% sodium dodecyl sulfate) containing a protease inhibitor cocktail and phosphatase inhibitors was used to extract total protein samples from cell pellets. For ODC, spermidine synthase (SRM), spermine synthase (SMS), spermidine/spermine N1-acetyltransferase (SAT1), polyamine oxidase (PAOX), and β-actin, a WES capillary Western system (12–230 kD Master kit α-Rabbit–HRP; PS-MK01; Protein Simple) was performed following instructions in the ProteinSimple user manual. In brief, protein samples were diluted with 0.1 × Sample Buffer to the concentration of 1.25 mg/mL. Protein samples and 5x Fluorescent Master Mix were mixed in a micro-centrifuge tube and heated at 95 °C for 5 min. After denaturation step, the samples, blocking reagent, primary antibodies (1:50 anti-ODC1 (ab97395, Abcam), 1:50 anti-SMS (HPA029852, Sigma-Aldrich), 1:50 anti-SRM (ABF257, Merck Millipore, Germany), 1:50 anti-SAT1 (ab105220, Abcam), 1:50 anti-PAOX (ab75119, Abcam), and 1:50 anti-β-actin (4967, CST, MA, USA), HRP-conjugated secondary antibodies and chemiluminescent substrate were dispensed into designated wells in an assay plate. A biotinylated ladder provided molecular weight standards for each assay. The data were analyzed using Compass software (Protein Simple).

### Chromatin immunoprecipitation

Chromatin samples were prepared by Auto iDeal chromatin immunoprecipitation sequencing (ChIP-Seq) kit for histones (C01010171, Diagenode, NJ, USA). Chromatin shearing was performed using Covaris S220 (Covaris Inc., MA, USA) in order to optimize the fragment size around 300 base pairs under the following conditions: 2% duty cycle, peak incident power of 105, 200 cycles per burst, and a 12 min program at 4 °C. Immunoprecipitation was performed using an SX-8G compact (Diagenode) with a direct ChIP mode. Polymerase II antibodies (ab5131, 1/50, Abcam, MA, USA) were used.

### ChIP-Seq data analysis

FASTQ sequences were aligned to the human hg19 genome sequence using Bowtie2^44^ and converted to sequence alignment map (SAM) and then binary alignment map files. Then, ChIP-Seq peaks were identified using MACS1.4^45^. Transcription start site (TSS) plots were generated using ngsplot (https://github.com/shenlab-sinai/ngsplot). The Bowtie-aligned peaks and model-based analysis of ChIP-Seq (MACS)-determined peak positions were visualized using Integrative Genomics Viewer^46^.

### Quantification and statistical analysis

Differences between groups were presented as mean ±SD, as noted in the figures. Experimental sample numbers are indicated in the figures. Data were analyzed using Student’s *t* test for two groups. *p* < 0.05 was considered statistically significant.

### Accession numbers

The Gene Expression Omnibus accession numbers reported in this paper are GSE102052, GSE102053, and GSE103187.

## Electronic supplementary material

FigureS1

FigureS2

Supplementary Figure Legends

TableS1
