# Supplementary figures and images for: Polyamine flux suppresses histone lysine demethylases and enhances ID1 expression in cancer stem cells
Source: Cell Death Discov. 2018 Nov 13;4:104. doi: 10.1038/s41420-018-0117-7 (PMC6234213; doi:10.1038/s41420-018-0117-7)

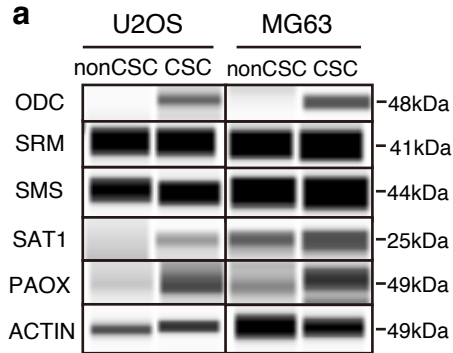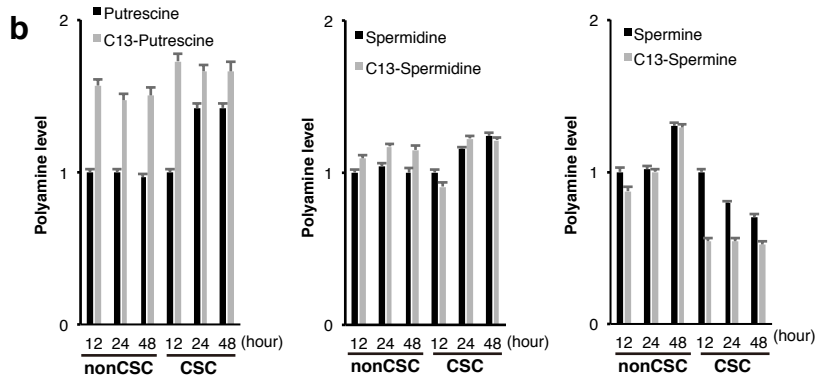

Supplement: Supplementary file 1 — FigureS1 [file 41420_2018_117_MOESM1_ESM.pdf]

**a**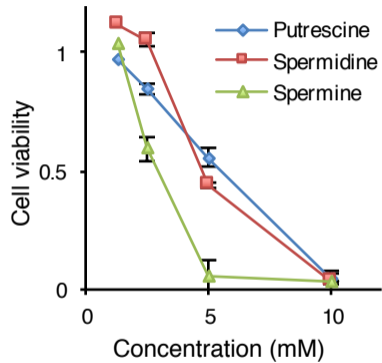**b**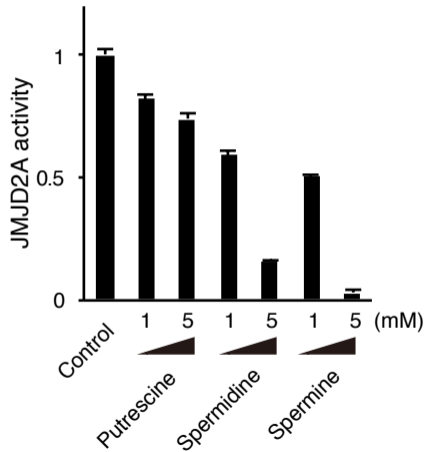**c**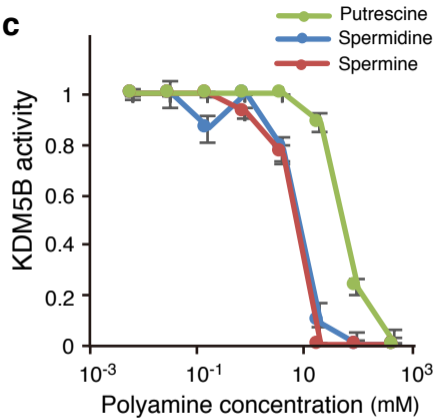

Supplement: Supplementary file 2 — FigureS2 [file 41420_2018_117_MOESM2_ESM.pdf]
