# Supplementary material for: Polyamine flux suppresses histone lysine demethylases and enhances ID1 expression in cancer stem cells
Source: Cell Death Discov. 2018 Nov 13;4:104. doi: 10.1038/s41420-018-0117-7 (PMC6234213; doi:10.1038/s41420-018-0117-7)
Supplement: Supplementary file 3 — Supplementary Figure Legends [file 41420_2018_117_MOESM3_ESM.docx]

Fig. S1. CSCs have considerable ODC and rapidly convert ornithine to PAs. (*A*) Immunoblot for ODC, SRM, SMS, SAT1, PAOX and actin using CSCs and non-CSCs from U2OS and MG63 cells. (*B*) A C13-ornithine flux analysis by GC-MS with HeLa cells collected at 12, 24, and 48 h. Data are represented as mean ± SD. n = 4.

Fig. S2. PAs can inhibit H3K4 demethylases. (*A*) A cell toxicity test using putrescine, spermidine, and spermine for 48 h exposure. Data are represented as mean ± SD. n = 3. (*B*) Enzymatic activity assay of JMJD2A. The demethylase activity was normalized by water (control). Data are represented as mean ± SD. n = 3. (*C*) The enzymatic activity assay of KDM5B. The demethylase activity was normalized by water (control). Data are represented as mean ± SD. n = 3.
