# Supplementary material for: Polyamine flux suppresses histone lysine demethylases and enhances ID1 expression in cancer stem cells
Source: Cell Death Discov. 2018 Nov 13;4:104. doi: 10.1038/s41420-018-0117-7 (PMC6234213; doi:10.1038/s41420-018-0117-7)
Supplement: Supplementary file 4 — TableS1 [file 41420_2018_117_MOESM4_ESM.docx]

| Primers | SOURCE | IDENTIFIER |
| --- | --- | --- |
| ODC1 fwd  CCCAGCGTTGGACAAATACT | Sigma | N/A |
| ODC1 rev  TCCATAGACGCCATCATTCA | Sigma | N/A |
| ID1 fwd  AATCATGAAAGTCGCCAGTG | Sigma | N/A |
| ID1 rev  ATGTCGTAGAGCAGCACGTTT | Sigma | N/A |
| ID2 fwd  ATGAAAGCCTTCAGTCCCGT | Sigma | N/A |
| ID2 rev  TTCCATCTTGCTCACCTTCTT | Sigma | N/A |
| ID3 fwd  TCATCTCCAACGACAAAAGG | Sigma | N/A |
| ID3 rev  ACCAGGTTTAGTCTCCAGGAA | Sigma | N/A |
| LSD1 fwd  ATCTGCAGTCCAAAGGATGG | Sigma | N/A |
| LSD1 rev  GCCAACAATCACATCGTCAC | Sigma | N/A |
| ACTB fwd  AGAGCTACGAGCTGCCTGAC | Sigma | N/A |
| ACTB rev  AGCACTGTGTTGGCGTACAG | Sigma | N/A |
| ID1 promoter fwd  TACAGTGCCCGCTGTACAAA | Sigma | N/A |
| ID1 promoter rev  GGGAAAAAGGCCTGAGTTCT | Sigma | N/A |
| ID2 promoter fwd  GATCATGGGAGAAGGCACTG | Sigma | N/A |
| ID2 promoter rev  CGCATTCCTAGCTTTTTCCA | Sigma | N/A |
| ID1 *XhoI* fwd  CTACTCGAGCACCATGAAAGTCGCCAGTGGCAG | Sigma | N/A |
| ID1 *NotI* rev  GCAGCGGCCGCTCAGCGACACAAGATGCGATC | Sigma | N/A |
| ID2 *XhoI* fwd  CTACTCGAGCACCATGAAAGCCTTCAGTCCCGTG | Sigma | N/A |
| ID2 *NotI* rev  GCAGCGGCCGCTCAGCCACACAGTGCTTTGC | Sigma | N/A |

**Table S1**. List of primer sequences for RT-qPCR.
